# 4-Deoxyaurone Formation in *Bidens ferulifolia* (Jacq.) DC

**DOI:** 10.1371/journal.pone.0061766

**Published:** 2013-05-08

**Authors:** Silvija Miosic, Katrin Knop, Dirk Hölscher, Jürgen Greiner, Christian Gosch, Jana Thill, Marco Kai, Binita Kumari Shrestha, Bernd Schneider, Anna C. Crecelius, Ulrich S. Schubert, Aleš Svatoš, Karl Stich, Heidi Halbwirth

**Affiliations:** 1 Institut für Verfahrenstechnik, Umwelttechnik und Technische Biowissenschaften, Technische Universität Wien, Wien, Austria; 2 Institut für Organische Chemie und Makromolekulare Chemie (IOMC, Lehrstuhl II/Schubert), Friedrich-Schiller Universität of Jena, Jena, Germany; 3 Max-Planck-Institut für chemische Ökologie, Jena, Germany; Lawrence Berkeley National Laboratory, United States of America

## Abstract

The formation of 4-deoxyaurones, which serve as UV nectar guides in *Bidens ferulifolia* (Jacq.) DC., was established by combination of UV photography, mass spectrometry, and biochemical assays and the key step in aurone formation was studied. The yellow flowering ornamental plant accumulates deoxy type anthochlor pigments (6′-deoxychalcones and the corresponding 4-deoxyaurones) in the basal part of the flower surface whilst the apex contains only yellow carotenoids. For UV sensitive pollinating insects, this appears as a bicoloured floral pattern which can be visualized in situ by specific ammonia staining of the anthochlor pigments. The petal back side, in contrast, shows a faintly UV absorbing centre and UV absorbing rays along the otherwise UV reflecting petal apex. Matrix-free UV laser desorption/ionisation mass spectrometric imaging (LDI-MSI) indicated the presence of 9 anthochlors in the UV absorbing areas. The prevalent pigments were derivatives of okanin and maritimetin. Enzyme preparations from flowers, leaves, stems and roots of *B. ferulifolia* and from plants, which do not accumulate aurones e.g. *Arabidopsis thaliana*, were able to convert chalcones to aurones. Thus, aurone formation could be catalyzed by a widespread enzyme and seems to depend mainly on a specific biochemical background, which favours the formation of aurones at the expense of flavonoids. In contrast to 4-hydroxyaurone formation, hydroxylation and oxidative cyclization to the 4-deoxyaurones does not occur in one single step but is catalyzed by two separate enzymes, chalcone 3-hydroxylase and aurone synthase (catechol oxidase reaction). Aurone formation shows an optimum at pH 7.5 or above, which is another striking contrast to 4-hydroxyaurone formation in *Antirrhinum majus* L. This is the first example of a plant catechol oxidase type enzyme being involved in the flavonoid pathway and in an anabolic reaction in general.

## Introduction

Flower colour is a result of the evolutionary development from undirected pollination by wind to directed pollination by a specific vector [1]–[4]. Yellow coloration appeared as an adaptation to the colour sense of insects as the prevalent pollinators in temperate zones [2]. Many Asteraceae species accumulate two types of yellow pigments, carotenoids and anthochlors. In *Bidens* sp., the carotenoids are uniformly distributed across the petal whereas the anthochlors are concentrated at the petal base [5], [6]. Such patterns are known as nectar guides (synonym: honey guides, pollen guides) [7]. For humans, the flowers are monochromatically yellow; however for ultraviolet (UV) sensitive insects the flowers appear bicoloured because of the different UV absorbance of carotenoids and anthochlors [1], [8]. *Bidens ferulifolia* is an interesting model plant for studying both nectar guide formation and anthochlor biosynthesis, which is not completely understood so far [9].

**Figure 1:**
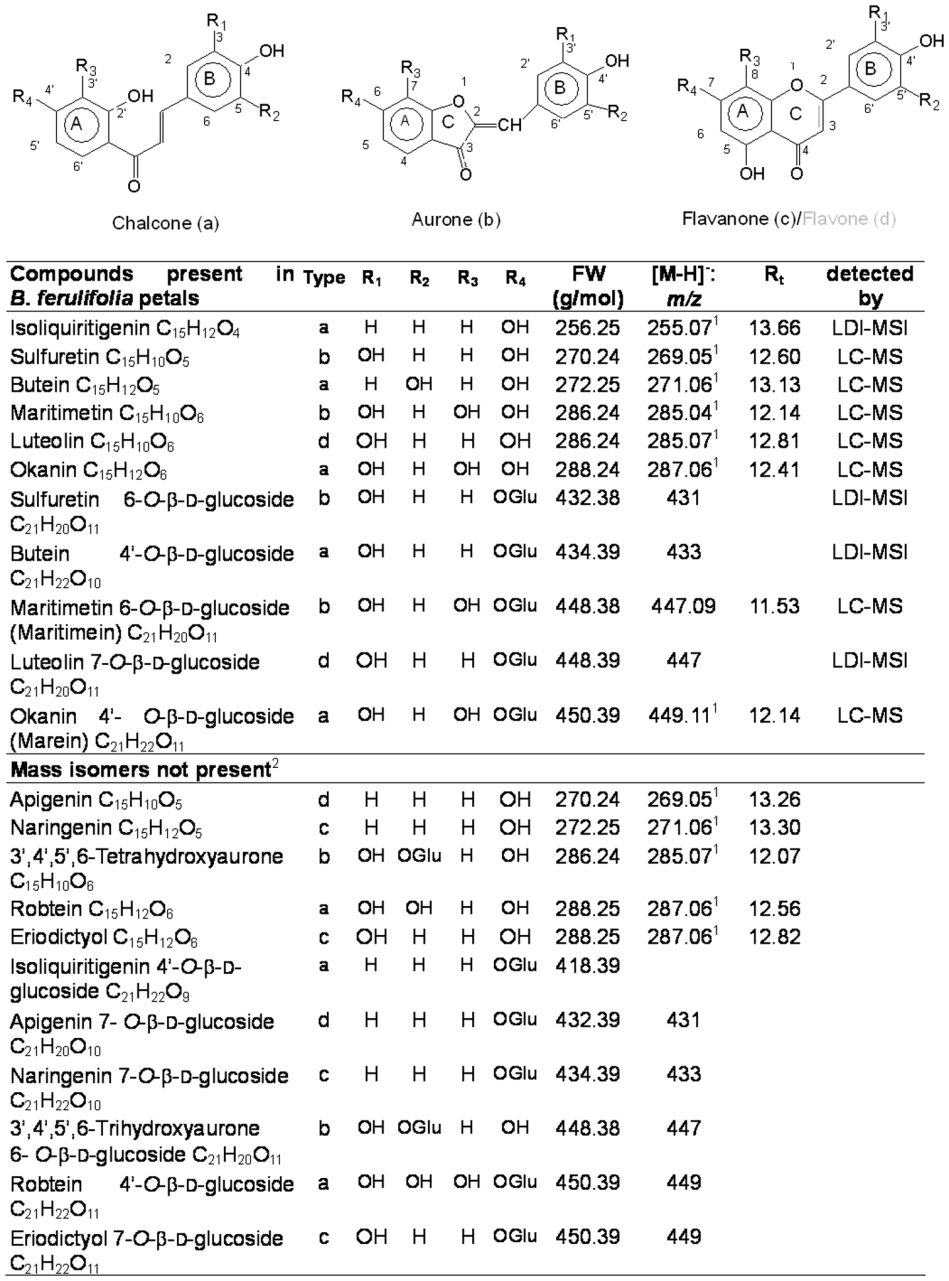
Anthochlors and flavonoids present or absent in *Bidens ferulifolia* petals. Please note the divergent ring numbering. ^1^exact mass obtained by LC-MS, ^2^ presence excluded by LC-MS.

Although the occurrence of anthochlors (chalcones and aurones) is not restricted to flower tissues they are mostly noticed as the yellow flower pigments in Asteraceae species, snapdragon (*Antirrhinum majus* L.) and carnations (*Dianthus caryophyllus* L.) [10], [11]. Anthochlors are frequently ranked among flavonoids, but their structure cannot be derived from the flavonoid skeleton (Figure 1). Hydroxy types and deoxy types are distinguished, depending on whether or not the position 6′ of chalcones and the corresponding position 4 of aurones carry a hydroxyl group. In Asteraceae, anthochlor pigments of the deoxy type are typically found with the exception of *Helichrysum bracteatum* which accumulates the hydroxy type pigments as *Antirrhinum majus* [10]. Whereas 6′-hydroxychalcones rapidly isomerise - either chemically or enzymatically - to the corresponding flavanone, the 6′-deoxychalcones are chemically stable and are not converted by common chalcone isomerases (CHI). Anthochlors are easily detected by a specific colour switch from yellow to orange when exposed to ammonia or the alkaline vapour of a cigarette [10]. This is due to the pH dependent transition of the undissociated phenol groups to phenolates, which results in a bathochromatic shift of approx. 100 nm from the violet to the blue range of the spectrum (Figure S1). The corresponding shift of the reflected wavelengths is perceived as colour switch by the human eye.

In contrast to the well-studied flavonoid pathway, the knowledge on the formation of anthochlors and particularly of aurones is still limited. The formation of aurones competes with flavonoid formation for chalcones as the immediate biochemical precursors. Originally, it was assumed that peroxidases could be involved [12] and actually peroxidase cDNA clones were isolated from cell cultures of *Medicago truncatula*, which converted chalcones to aurones as a response to yeast elicitor [13]. In addition, fungal catechol oxidases can catalyze aurone formation [14], [15]. However, during the past decade it was shown that a soluble bifunctional polyphenol oxidase (PPO) homologue (monooxygenase and dioxygenase activity) is involved in the formation of aurone based flower pigments, but studies concentrated exclusively on *A. majus* [15]–[19], which accumulate the less common hydroxy type anthochlors. Aureusidine synthase from *A. majus*, catalyzes the hydroxylation and oxidative cyclization of chalcones to aurones. In the suggested reaction mechanism [20], the monoxygenase activity is particularly crucial to explain the occurring 3′,4′- and 3′,4′,5′-hydroxylation pattern of the aurones present in *A. majus*. In addition, it is essential for the conversion of the monohydroxylated naringenin chalcone, because oxidative cyclization occurs only in the presence of hydroxyl groups in *ortho*-position. Aureusidine synthase accepted also 6′-deoxychalcones although they are not native substrates in *A. majus*. Thus, it was assumed that formation of 4-deoxyaurones in Asteraceae species follows a similar mechanism [20]; however, this was not tested so far in any 4-deoxyaurone producing plant.

Using a combined approach of mass spectrometry, biochemical assays, and UV photography, we investigated for the first time the formation of 4-deoxyaurones in *Bidens ferulifolia* (Jacq.) DC. We show that the hydroxylation step of the B-ring requires the presence of an additional hydroxylating enzyme. In addition, we demonstrate for the first time that the ability of aurone formation is frequently present in plants and not necessarily correlated to aurone accumulation, thereby contributing a novel perspective to the knowledge of aurone biosynthesis in general.

## Results

### Anthochlors and flavonoids in *Bidens ferulifolia* petals

The presence of anthochlor pigments and flavonoids in the *Bidens* petals was analysed by a combined approach of matrix-free UV laser desorption/ionisation mass spectrometric imaging (LDI-MSI), liquid chromatography-mass spectrometry (LC-MS) and high-performance liquid chromatography (HPLC) with emphasis on identifying the chalcone and aurone core structures. LDI-MSI demonstrated the presence of 9 mass peaks corresponding to 6 core structures of anthochlor pigments or flavonoids and the corresponding monoglucosides, respectively (Figure 1). A mass peak related to isoliquiritigenin 4′-*O*-ß-d-glucoside (*m/z* 417) was not detected. Mass peaks *m/z* 269, 271, 285, 287, 431, 433, 447 and 449 were not clearly related to a specific compound because the presence of several mass isomers could be expected (Figure 1). The compounds were further identified by LC-MS measurement of exact mass and retention time in comparison to commercially available reference compounds (Figure 1). The native methanolic extracts contained anthochlors as aglycones and glycosides. The latter were not identified further because the glucosylation pattern of the pigments is not decisive for the nectar guide formation. The presence of those glycosides, which were not available as commercial references, was investigated after enzymatic hydrolysis and the glucose position was tentatively allocated according to the literature [10], [21]. Thus, the LDI-MSI mass peaks could be allocated to specific compounds as follows: *m/z* 269 (sulfuretin), *m/z* 271 (butein), *m/z* 285 (maritimetin/luteolin), *m/z* 287 (okanin), *m/z* 431 (sulfuretin 6-*O*-ß-d-glucoside), *m/z* 433 (butein 4′-*O*-ß-d-glucoside), *m/z* 447 (maretimein/luteolin 7-*O*-ß-d-glucoside) and *m/z* 449 (marein). The presence of 3′,4′,5′,6-tetrahydroxyaurone and robtein and their glycosides could be excluded (Figure 1). The abundance of the main pigments was analyzed by HPLC. After enzymatic hydrolysis of methanolic extracts, okanin (2.6 mg±0.5/10 g fresh weight) followed by maritimetin (1.3 mg±0.7/10 g fresh weight) and butein (0.4 mg±0.3/10 g fresh weight) were the prevalent anthochlor pigments. High amounts of the flavone luteolin (1.4 mg±0.4/10 g fresh weight) were also present.

### 4-Deoxyaurone formation in *B. ferulifolia*

**Figure 2:**
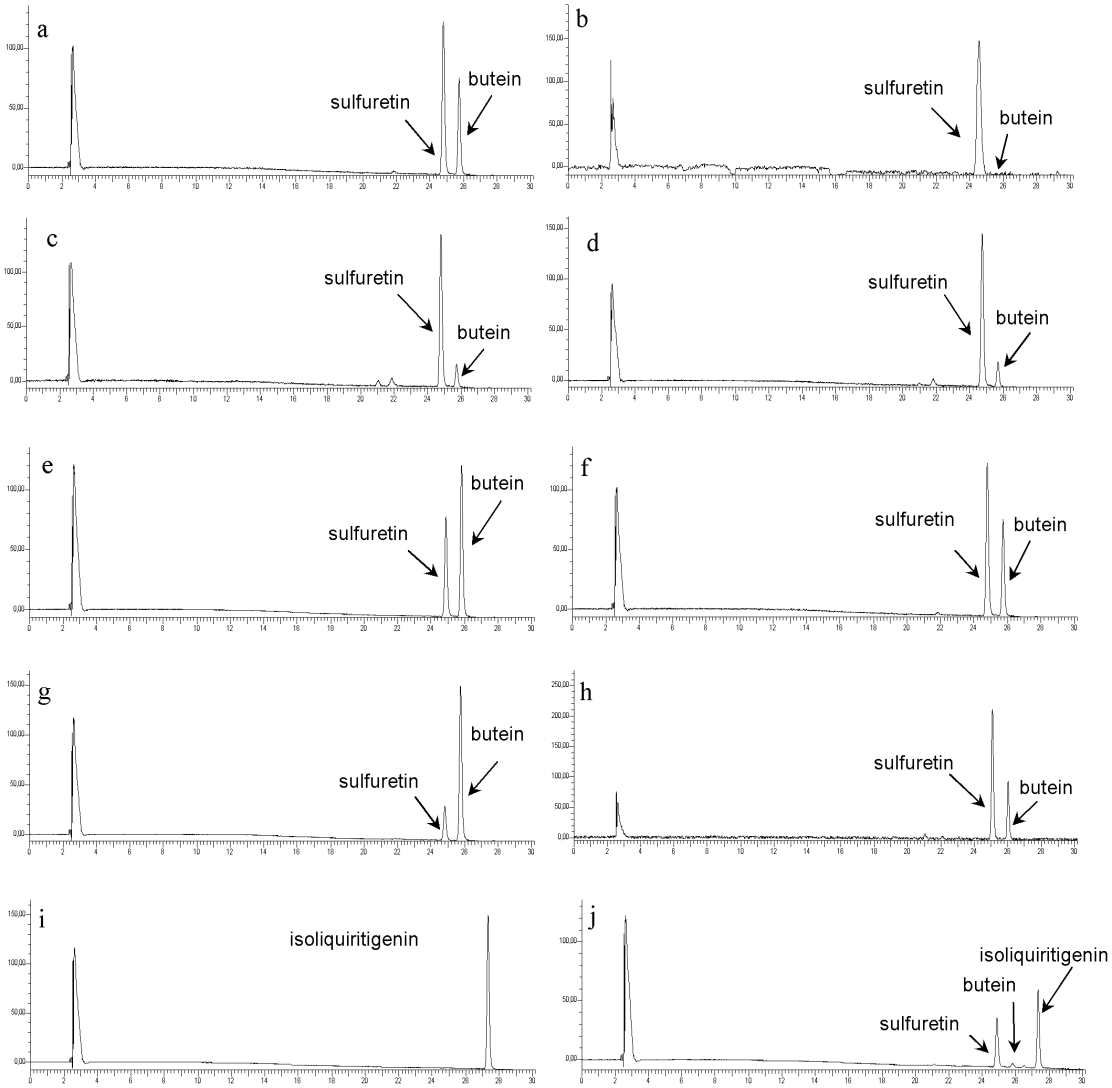
HPLC chromatograms from incubation of enzyme preparations. *Bidens ferulifolia* petals (a) and leaves (b), *Antirrhinum majus* petals (c) and leaves (d), *Arabidopsis thaliana* col-0 plants (e), *Tagetes erecta* petals (f), *Dianthus caryophyllus* petals (g), and *Petunia hybrida* petals (h) with butein and of enzyme preparations from *B. ferulifolia* petals (i) and *Antirrhinum majus* petals (j) with isoliquiritigenin.

Incubation of butein with enzyme preparations from *B. ferulifolia* petals led to the formation of one product which was identified as sulfuretin by comparison with the authentic reference compound (Figure 2 a). No formation of robtein or 5′-hydroxysulfuretin could be observed under standard conditions. Apart from butein, the enzyme accepted the 6′-deoxychalcones okanin and robtein as substrates and converted them into the corresponding aurones maritimetin and 5′-hydroxysulfuretin. Butein 4′-*O*-glucoside was converted into sulfuretin 6-*O*-glucoside accordingly. Hydroxylating activity in addition to the ring closure could be observed in none of the assays. When marein was offered as a substrate, the formation of okanin and the corresponding aurone maritimetin could be detected. No product formation could be observed when isoliquiritigenin or naringenin chalcone were offered, neither under standard conditions nor in the presence of 3-[(3-cholamidopropyl)dimethyl-ammonio]-1-propanesulfonate (CHAPS) and/or hydrogen peroxide (H_2_O_2_). Eriodictyol chalcone was also converted to aureusidine but was an unsuitable substrate for enzyme characterisation due to chemical instability in both acidic and alkaline environment.

The ability of aurone formation was tested with enzyme preparations from various *B. ferulifolia* tissues including those which do not accumulate aurones. Conversion of chalcones into aurones was catalyzed by enzyme preparations from flower tissues, leaves, stems and roots (Table S1). Flower tissues included petals from disc florets and 3 developmental stages of ray florets and sepals. Highest specific activities were observed in roots followed by open flowers; lowest activities were found in leaves. However on a fresh-weight basis, activity in petals and leaves were higher (Table S1). The presence of ascorbate strongly influenced the AUS activity. When no ascorbate was present in the buffer during the enzyme preparation, neither the substrate nor the product could be detected in the assay. The presence of ascorbate concentrations up to 0.02 µM during enzyme preparation did not affect product formation. At higher concentrations, the AUS activity decreased steadily. Aurone formation showed a high dependence on substrate concentration. Thus, highest activities were observed in the presence of a substrate concentration as low as 0.5 mM and decreased steadily with increasing concentrations. However, product formation could be also observed in the presence of 30 mM.

Using butein as a substrate, the reaction showed highest activities at pH 7.5. At lower pH values, the activity decreased rapidly, at higher pH values slowly. At pH 5.5, 25% of the activity compared to the pH optimum was observed (Table 1). pH Optima for okanin, robtein and butein 4′*O*-glucoside were slightly higher (Table 1). Eriodictyol chalcone was converted at acidic and alkaline conditions. Quantification, however, was not possible because of substrate instability. The reaction was characterized in more detail using butein as substrate. Highest reaction rates were observed at 25°C. At 60°C, reaction rates still reached 21% of the values measured at 25°C; at temperatures higher than 60°C no AUS activity could be observed. In the presence of heat inactivated preparations, no product formation was observed at any pH or temperature tested. The reaction was linear with time up to 15 min and with protein concentrations up to 3 µg. The values for apparent K_m_ and V_max_ for butein were 113 µM and 387 µmol/s*kg protein respectively. The ratio V_max_/K_m_ was 3.4 thus confirming butein as natural substrate.

**Table 1:** Dependence of the 4-deoxyaurone formation on pH.

Substrate	pH optimum	activity at optimal pH (mol/s.g)	relative activity at pH 7.0 [%]	relative activity at pH 5.5 [%]
butein	7.50	0.4±0.1	97	25
okanin	8.50	2.4±0.2	18	11
robtein	8.50	0.5±0.05	18	9
butein 4-*O*-glucoside	8.25	0.01±0.005	42	14

Relative activities were calculated in comparison to the activity at the optimal pH which was defined as being 100%. Substrate preference cannot be concluded from comparison of the activities.

### Flavonoid metabolism in *Bidens* petals

**Figure 3:**
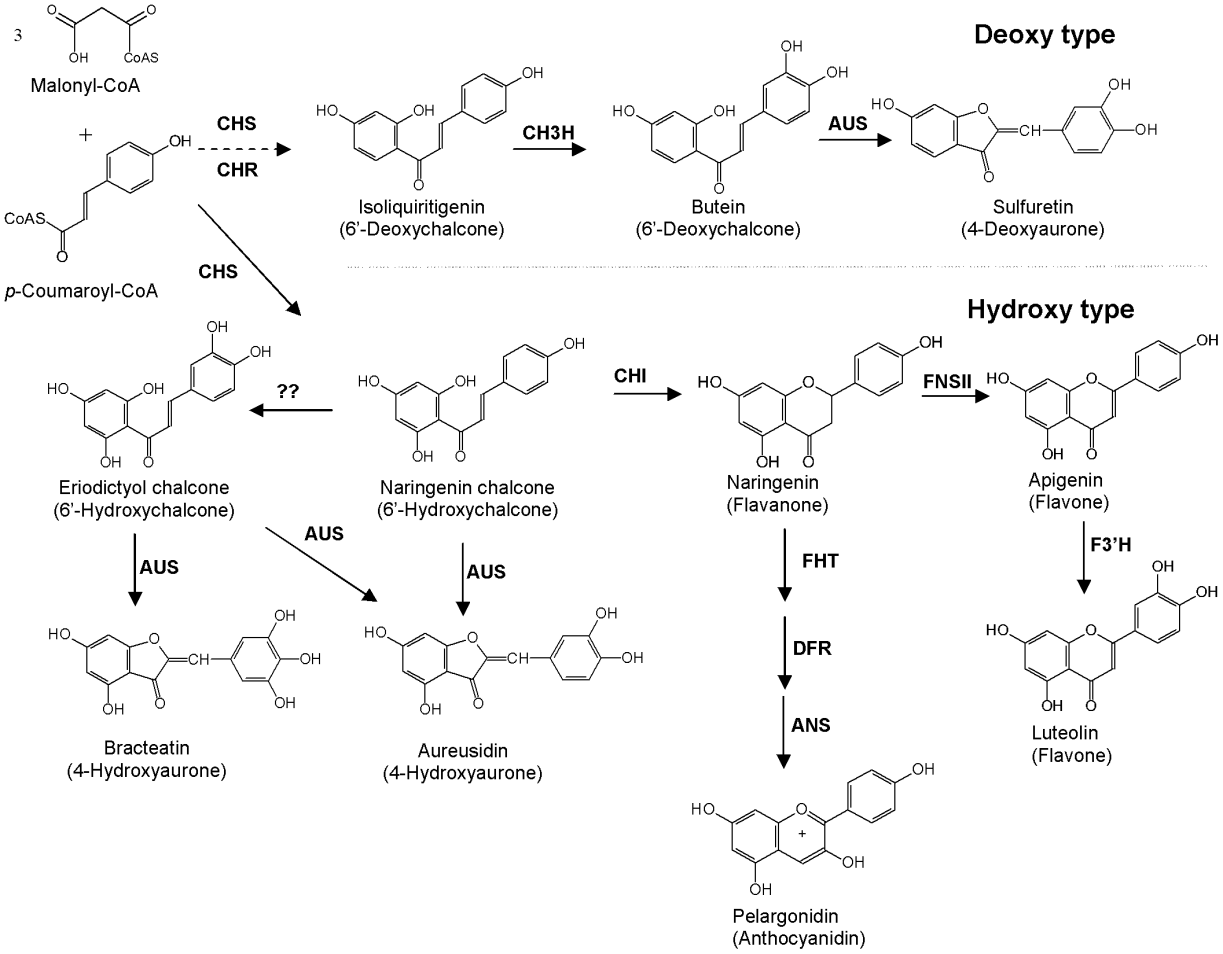
Overview on flavonoid and anthochlor formation from *p*-coumaroyl-CoA. abbrev.: ANS: anthocyanidin synthase, AUS: aurone synthase, CH3H: chalcone 3-hydroxylase, CHI: chalcone isomerase, CHR: chalcone reductase, CHS: chalcone synthase, DFR: dihydroflavonol 4-reductase, FHT: flavanone 3-hydroxylase, F3′H: flavonoid 3′-hydroxylase, FNSII: flavone synthase II.

Enzyme preparations from petals of *B. ferulifolia* were analysed for the presence of enzyme activities of the pathways leading to anthochlors and flavonoids (Figure 3). The activities of chalcone synthase/chalcone isomerase (CHS/CHI), flavanone 3-hydroxylase (FHT), flavone synthase II (FNS II), AUS, flavonoid 3′-hydroxylase (F3′H), and chalcone 3-hydroxylase (CH3H) could be detected in the petals (Table S2). Dihydroflavonol 4-reductase (DFR) activity was not found. A type II CHI activity, which catalyzes the isomerisation of 6′-deoxychalcones to the corresponding 5-deoxyflavanones [22] was not detected either. The presence of a chalcone reductase (synonyms: polyketide reductase, chalcone ketide reductase) [23] activity (CHR) could not be demonstrated. All enzymes detected showed higher activities in the petal base compared to the apex but were clearly present in both parts.

### 4-Deoxyaurone formation with enzyme preparations from further plants

Enzyme preparations from further plants which do not naturally accumulate anthochlorspigments, were also able to catalyze the conversion of 6′-hydroxychalcones and 6′-deoxychalcones to the corresponding aurones. Due to the instability of eriodictyol chalcone as substrate only the chromatograms with butein are shown (Figure 2). This included a number of other ornamental plants from the Asteraceae family e.g. *Tagetes erecta* and *Rudbeckia hirta*, but also members of other plant families e.g. *A. majus*, *Petunia hybrida*, *D. caryophyllus* and *A. thaliana* (Figure 2). In *A. majus*, AUS activity was present in enzyme preparations from petals and leaves (Figure 2 c, d). *B. ferulifolia* and *A. majus* showed a striking difference in the acceptance of isoliquiritigenin as a substrate (Figure 2 i, j). Enzyme preparations from *A. majus* catalyzed the formation of butein and sulfuretin from isoliquiritigenin. Addition of H_2_O_2_ or CHAPS was not required. The presence of KCN strongly decreased the formation of butein and sulfuretin whereas the cytochrome P450 specific inhibitors ketoconazol and ancymidol did not. Enzyme preparations from *B. ferulifolia*, in contrast, converted isoliquiritigenin to butein only in the presence of NADPH. The other plants did not convert isoliquiritigenin, neither in the presence nor in the absence of NADPH, which can be explained by the restricted occurrence of chalcone 3-hydroxylase activity [24].

### Nectar guides

**Figure 4:**
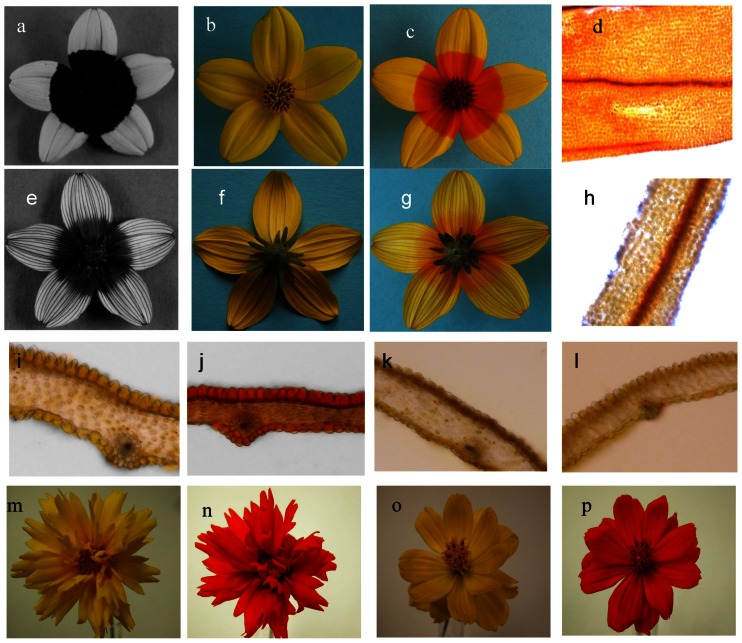
UV nectar guides. *Bidens ferulifolia* flower UV photography of front (a) and back (e) side, daylight photographies (b, f) and after (c, g) ammonia staining. Cross sections of *B. ferulifolia* petals base native (i) and stained (j) and petal apex native (k) and stained (l). Epi-illumination mode microscopic view of stained epidermis of petal front side base (d) and apex (h). *Coreopsis grandiflora* flower before (m) and after (n) ammonia staining. *Cosmos sulphureus* flower before (o) and after (p) ammonia staining.

UV photography of *B. ferulifolia* showed the presence of the typical bulls-eye, which is composed of a UV absorbing petal base and a UV reflecting apex (Figure 4 a). The back side, in contrast, showed a different pattern with a faintly UV absorbing centre and UV absorbing rays along the otherwise UV reflecting petal apex (Figures 4 e). Staining with ammonia led to a colour switch of the petal base in the same areas (Figure 4 b, c, f, g), indicating that the UV nectar guides are based on the local accumulation of anthochlors as described earlier [6]. Particularly the veins were intensively stained along the whole petal (Figure 4 d, g, h). The anthochlors are present in the epidermal layer of both sides of the petal base (Figure 4 i, j), whereas in the cross sections of the apex only the veins at the back side were stained (Figure 4 g, k, l). Petals from all developmental stages showed the typical colour switch after ammonia staining. However, petals of closed buds were two times folded and only the back side was visible at the early stages of flower anthesis. In contrast to *B. ferulifolia, Coreopsis grandiflora* Hogg ex Sweet and *Cosmos sulphureus* Cav showed a complete colour switch demonstrating the uniform distribution of anthochlors in their petals (Figure 4 m–p).

**Figure 5:**
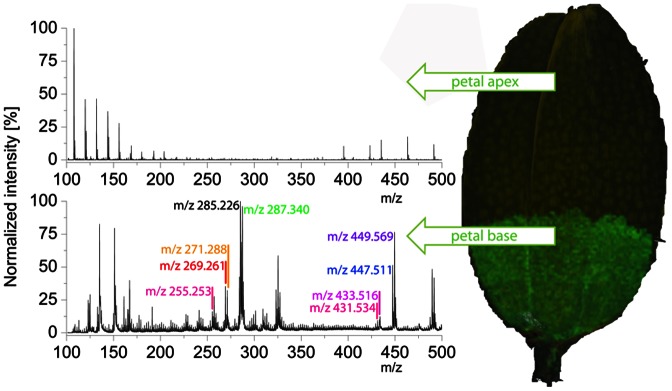
LDI-MS of a *Bidens ferulifolia* petal. a: a representative spectrum of the petal base containing nine *m/z* signals of anthochlors. b: no anthochlors were detected in representative spectra of the petal apex. For the allocation of *m/z* signals to compounds refer to Figure 1.

**Figure 6:**
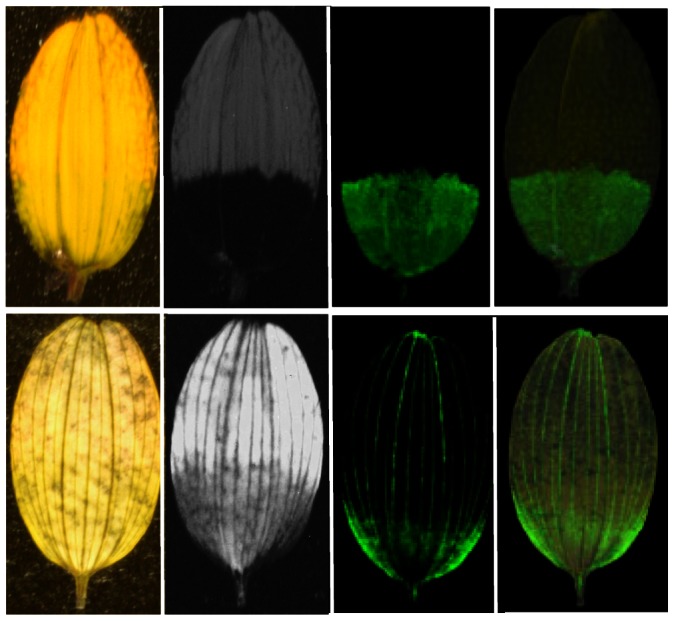
Spatial distribution of okanin (*m/z* 287) along a *Bidens ferulifolia* petal fixed by adhesive tape on a Indium Tin Oxide glass slide. first row: front side, second row: back side; from left to right: daylight photos, UV photos, negative ion mode LDI-MSI images (green area indicates the presence of the target compound), negative ion mode LDI-MSI images overlapping the daylight photos. All mass peaks related to anthochlors showed a similar distribution.

In the LDI-MS image, all 9 mass peaks showed a similar distribution along the petal and were exclusively present at the base but not in the apex of the petals front side (Figure 5, 6). LDI-MSI was also possible when the petals were stained with ammonia, however, this negatively affected leaf quality, with the exception of imaging of the veins. It appears that the ammonia might soften the relatively stiff structure of the veins thereby facilitating the extraction of the analytes. All mass peaks were found on both petal sides with the exception of the mass peaks *m/z* 433 and *m/z* 431, which were present only on the front side.

## Discussion

The ornamental plant *B. ferulifolia* is an easily accessible model for studying UV nectar guides because a rapid visualization by ammonia staining instead of UV photography, which needs specialized equipment, is possible. In addition, *B. ferulifolia* offers the possibility to study for the first time the biosynthesis of aurones in plants, which accumulate the deoxy type anthochlors as flower pigments. Studies on the deoxy type pathway have the advantage that the chemically stable 6′-deoxychalcones are the native substrates for aurone formation and no competition for common precursors for flavanone formation by CHI occurs. We preferred *B. ferulifolia* over other Asteraceae species as model plant because the irregular distribution in the petals and the striking presence particularly in veins offers interesting possibilities for future studies on the regulation and tissue or cell specific formation of anthochlors in the flowers.

### 4-Deoxyaurone formation

The presence of 9 compounds belonging to the class of anthochlor pigments was observed. Surprisingly 5 of them were aglycones although in the literature the corresponding glycosides are reported [6]. Thus, it can be concluded that aurone formation *in planta* actually occurs at the aglycone level and precedes the glucosylation step. However, it is possible that aurone formation also takes place at the level of chalcone glycosides because glucosylated chalcones were accepted as substrates as well. Whereas the presence of compounds based on isoliquiritigenin, butein, sulfuretin, okanin and maritimetin was expected for *Bidens* sp., the presence of anthochlors carrying 3 hydroxyl groups in the B-ring was not reported and was not demonstrated in our studies either. This allows important clues on the mechanism of 4-deoxyaurone formation, because it underpins that AUS does not introduce an additional hydroxyl group in the B-ring as part of the catalytic mechanism as is the case in 4-hydroxyaurone formation (Figure 3).

Studies on the formation of aurone based flower pigments has concentrated on the 4-hydroxyaurone accumulating *A. majus* so far and was shown to be catalyzed by the so-called ‘aureusidine synthase’ [15]. However, we favour the more general term ‘aurone synthase’ (AUS), because the enzyme catalyzes the formation of both aureusidine and bracteatin from eriodictyol chalcone and also accepts other chalcone substrates. This is also in better agreement with the physiological activity in Asteraceae where aureusidine formation is not native. In *B. ferulifolia*, AUS activity was present in different flower tissues including non-aurone accumulating sepals, but also in roots, stems and leaves. Furthermore, aurone formation could be demonstrated with enzyme preparations from a number of plants which do not naturally accumulate aurones e.g. *A. thaliana*. Thus, the presence of AUS activity is not specifically correlated with aurone occurrence. One possible explanation is that aurone formation is catalyzed by an unspecific enzyme e.g. a PPO because AUS was classified as being a Cu^2+^ containing PPO homologue. This is supported by the fact that the suggested reaction mechanism is actually based on an enzymatic *o*-quinone formation, which is a classical PPO reaction, followed by a non-enzymatic cyclization step [25]. The argument against is that the purified aureusidine synthase specifically acted on chalcones whereas 3,4-dihydroxyphenylalanine (DOPA), which is one of the most common substrates for PPOs, was not accepted [15]. As the observed AUS activity accepted only substrates possessing a dihydroxyl pattern in the B-ring it maybe concluded that in contrast to the aureusidine synthase from *Antirrhinum majus*, AUS in the Asteraceae species is a catechol oxidase homologue rather than a tyrosinase homologue.

The assumption that unspecific enzymes can be involved in aurone formation is supported by the fact that recombinant peroxidases from *M. truncatula* were able to convert chalcones to aurones [13]. However accumulation was only found so far in cell cultures as a response to yeast elicitor and was interpreted as metabolic spillover after the accumulation of unusually high levels of chalcone precursors [13]. Aurone formation by peroxidase - and catechol oxidase type enzymes can be clearly distinguished by differences in the H_2_O_2_ – requirement, acceptance of monohydroxylated substrates and product formation. Hispidol formation takes exclusively place when a peroxidase is involved and was never observed by aureusidine synthase from *A. majus* (Sato *et al.* 2001) or enzyme preparations from *B. ferulifolia* and the other plant species included in this study regardless of whether or not H_2_O_2_ was present in the assay.

Despite the high AUS specific activity observed, the ability of aurone formation in non-aurone accumulating plants and tissues is clearly not of physiological relevance and could be also due to the presence of PPOs, particularly from chloroplasts. However, leave tissues which usually show high chloroplast concentrations did not show a particularly high AUS activity. In addition, it is obvious that enzyme preparations from *B. ferulifolia* petals, in which aurone formation is of physiological relevance, did not show a specifically different or higher AUS specific activity than the other tissues and the observed substrate acceptance and product formation was in accordance with the anthochlors identified in the petals. Davies *et al.* [9] showed that AUS expression in different *A. majus* lines did not correlate with aurone formation and suggested the presence of a multigene family.

An explanation for the absence of aurones in most plants despite the common presence of a PPO type AUS activity is that aurone formation can occur only in a special biochemical background. The formation of 4-deoxyaurones requires a 6′-deoxychalcone precursor, which is provided by a rare CHR (synonyms: polyketide reductase, chalcone ketide reductase) found only in few plants so far [23], [26]. The immediate biochemical precursors of 4-hydroxyaurones, in contrast, are commonly formed in ornamental species [27] but are rapidly converted to downstream flavonoid products. Thus, 6′-hydroxychalcones are only found in mutants lacking the activities of CHI and of at least one further enzyme in the flavonoid pathway [28]. Thus, in the competition between flavanone and aurone formation velocity of the involved enzymes and further unknown factors must be decisive. AUS based biotechnological approaches to introduce yellow flower were not really convincing so far [18], [29]. It appears that a careful selection of the parental line is essential.

Our studies have identified several crucial differences between the aurone formation in *A. majus* and *B. ferulifolia*. Most important is the lack of the monooxygenase activity during the reaction regardless which 6′-deoxychalcone substrate was used. Thus, butein was converted to sulfuretin only, and isoliquiritigenin was not accepted as substrate. This is supported by the fact that the presence of robtein and tetrahydroxychalcone in *Bidens* sp. has never been reported in literature and could also not be demonstrated in our investigations. Thus, the presence of two vicinal hydroxy groups in the chalcone precursor is indispensable, which must be introduced by a separate enzyme. Hydroxylation of chalcones, which was described for the first time for *Dahlia variabilis* [30] could also be demonstrated with the enzyme preparations from *B. ferulifolia* petals (Table S2). Recently, it was shown that a specific CH3H is responsible which is not generally found in plants [24], [31]. This is in clear contrast to 4-hydroxyaurone formation where hydroxylation and cyclization can be catalyzed in one single step [19] (Figure 3). This is also supported by the fact that butein formation from isoliquiritigenin by enzyme preparations from *A. majus* decreased in the presence of the PPO inhibitor KCN whereas cytochrome P450 specific inhibitors had no influence.

A second important difference was the dependence of the reaction on pH. Highest activities were observed at alkaline conditions. Even though the observed optima for all substrates except butein were above physiological relevance, the activity was still high at pH 7.0, but low in the range of pH 5.0–6.5, where aureusidine synthase from *A. majus* showed its pH maximum [19]. From the observed pH optima it is more likely that 4-deoxyaurone formation takes place in the cytosol and not in the vacuoles as shown for 4-hydroxyaurone formation in *A. majus* [16]. The observed effect of ascorbate is typical for a PPO homologue. The mechanism is either based on reduction of oxidized polyphenol products or on enzyme inactivation if polyphenol substrates are absent [32]. Thus, ascorbic acid is frequently used to prevent browning reactions by PPOs and peroxidases in biochemistry and food technology [33]. The fact that no product or substrate was found when the assays were performed in the absence of ascorbic acid could be explained by oxidation and polymerization of the compounds by unspecific peroxidases or PPOs in the absence of the oxidation protectant otherwise abundantly found in enzyme preparations [34].

### Nectar guides

Nectar guides are the most subtle visual signals of flowers aiming at ‘brand recognition’ and persistent attraction of individual pollinators to ensure directed and specific pollination of a plant species [35]. They are caused by the irregular spatial distribution of compounds with divergent light absorbance behaviour at the petal's front side. Depending on the absorbance spectrum of the pigments and the visual sense of the target pollinator such floral patterns can be invisible to the human's eye and are typically visualized by UV photography [7]. Although the occurrence of UV nectar guides was largely investigated [5], [36]–[38], in-depth studies on the chemical base are so far limited to the flavonol-based UV nectar guides in *R. hirta* [39], [40] where it was shown that the spatial distribution of specific flavonols rather than a +/− system of flavonols in general is creating the pattern. In *B. ferulifolia*, however, nectar guides are based on other pigments and ammonia staining clearly demonstrated the presence of a +/− system of anthochlor pigments on the petal front side. On the back side, in contrast, anthochlor pigments are found at both the base and apex, but are restricted to the veins in the apex. The typical bulls-eye pattern of the front side is established already in the earliest developmental stages but remains hidden to pollinators until the buds are open and the petals unfolded. Recently, LDI-MSI proved to be highly suitable for the localization of secondary metabolites in plant tissues even at single-cell resolution [41]. Our work demonstrates the suitability of this technique for the non-destructive investigation of (UV) colour patterns in plants providing information of the chemical composition and the local distribution simultaneously.

*B. ferulifolia* flowers are yellow due to the absence of anthocyanins as a result of lacking DFR activity. The enzyme activities required for the nectar guide formation could be detected in the base and apex of the petals. Because anthochlors are also present in the apex veins on the back side this was not surprising. However, it remains a puzzle in which way the nectar guides are formed. This requires a tight cell specific regulation, resulting in anthochlor pigment formation in the epidermis at the front and back side of the petal base whereas in the apex the pathway is exclusively activated in the veins. For anthocyanin formation in *A. majus*, it was recently shown that epidermal specific venation is defined by overlapping expression domains of two coregulators of the pathway [42].

## Conclusions

Our knowledge on aurone biosynthesis is still limited in many aspects including the question how tissue and cell specific formation is regulated. The formation of yellow 4-hydroxyaurone pigments in snapdragon (Plantaginaceae) by the tyrosinase type aureusidine synthase is one of the rare examples for the involvement of type-3-copper-proteins in plant biosynthesis. We showed that formation of closely related 4-deoxyaurone pigments in Asteraceae species is different by the involvement of a catechol oxidase type enzyme and that the presence of AUS activity is more common than originally assumed. This strongly indicates that the decisive evolutionary step for aurone accumulation rather concerned the provision of suitable chalcone precursors than the actual ring closure step. The involvement of a catechol oxidase homologue in aurone formation introduces a novel enzyme class in the flavonoid pathway. To our knowledge, this is the first catechol oxidase type enzyme involved in an anabolic reaction. The establishment of the 4-deoxyaurone pathway (Figure 3) is the first crucial step for future studies on aurone formation at the molecular level. Our studies have demonstrated the advantages of the 4-deoxyaurone system, particularly with respect to the chemical stability of the 6′-deoxychalcone substrates and the absence of immediate competition of CHI and AUS for the same precursors which allows directly studying aurone formation with enzyme preparations from different plant tissues. Thus, Asteraceae species could be an excellent model system to investigate various aspects of aurone formation including regulatory issues.

## Materials and Methods

### Plant material

The investigations were carried out on *Bidens ferulifolia* L. flowers purchased as potted plants (Bellaflora, Vienna, Austria, www.bellaflora.at) and cultivated in the experimental field of TU Wien in Vienna in summers 2008–2011. Three developmental flower stages were collected: buds (folded petals, 5 mm length), opening flowers (unfolding petals, 9 mm length) and open flowers (unfolded petals, 12 mm length). For standard assays and enzyme characterisation, petals from ray florets of buds were used. For comparing AUS synthase in different plant tissues, 5 plants were separated into root, leaf, stem and flower tissues. Petals of *R. hirta* L. cv. Indian Summer, *T. erecta* L. cv. Antigua F_1_ gelb *A. majus* L. cv. Sonnett F_1_ gelb, and *P. hybrida* cv. White Corso (all grown from seeds of Austrosaat, Vienna, Austria, www.austrosaat.at) were also collected from the experimental field, yellow and white carnations were purchased as cut flowers from a local flower shop. Seeds of *Arabidopsis thaliana* Col-0 were obtained from the European Arabidopsis Stock Centre NASC and cultivated at standard conditions.

### UV photography

For UV photography, flowers or petals were radiated with a Camag Reprostar II at 366 nm (Camag, Muttenz, Switzerland). Pictures were taken with a Nikon D100 digital camera and a UV Nikkor lens (f = 105 mm, 1∶4.5, Nikon, Vienna, Austria, http://www.nikon.at) together with the UV pass filter B+W 58 403 (Schneider, Bad Kreuznach, Germany, http://www.schneiderkreuznach.com) and are shown as monochromes.

### Microsocopy

Petals were treated as described [43]. The tissue was infiltrated with a series of glycerol-water dilutions (v/v 1∶10, 1∶5, 1∶1) for one hour each. After the infiltration process, cross-sections were sliced manually from the petals still embedded in the glycerol-water 1∶1. The slices were transferred to a microscope slide and were embedded either in water (native) or in a solution of 2 M ammonia in glycerol-water (v/v 1∶1). The preparations were examined under a Carl Zeiss Axio Scope A1 microscope (Zeiss, Vienna, Austria, http://www.zeiss.at) and pictures were taken using a Canon G10 (Canon, Vienna Austria, http://www.canon.at) connected to the microscope *via* a conversion Canon lens adapter LA-DC58K (Zeiss No. 426126).

### Chemicals

[^14^C]Isoliquiritigenin and [^14^C]butein were synthesised as described [44] from 4-hydroxy[ring-U-^14^C]benzaldehyde (33.1 MBq/mg) (Amersham International, UK, http://www.gelifesciences.com). Isoliquiritigenin, butein, sulfuretin, luteolin, marein, maritimein, luteolin 7-*O*-glucoside and eriodictyol chalcone were purchased from Extrasynthesis (Genay, France, http://www.extrasynthese.com), robtein from TransMIT-MPC (Giessen, Germany, http://www.plantmetachem.com). Okanin and maritimetin were obtained by enzymatic hydrolysis from marein and maritimein, respectively. Briefly, 20 µg of the glycoside dissolved in 20 µL ethanol were incubated for 30 min at 30°C with 10 µg naringinase (Sigma-Aldrich, Vienna, Austria, http://www.sigmaaldrich.com) in 80 µL 0.1 M McIlvaine buffer pH 5.5.

### LC-MS analysis

The LC-MS analysis was performed using the Ultimate 3000 series RSLC (Dionex, Sunnyvale, CA, USA, http://www.dionex.com) system and the Orbitrap XL mass spectrometer (Thermo Fisher Scientific, Bremen, Germany, http://www.thermo.com) equipped with an ESI source. HPLC was accomplished using the Acclaim C18 Column (150×2.1 mm, 2.2 µm; Dionex) at a constant flow rate of 300 µL/min using a binary solvent system: solvent A contains water with 0.1% formic acid (Roth, Karlsruhe, Germany, http://www.carl-roth.de) and solvent B contains acetonitrile (hypergrade for LC MS, Merck KGaA, Darmstadt, Germany, http://www.merckchemicals.com) with 0.1% formic acid. Ten µL were injected into the HPLC gradient system started with 0.5% B and linearly increased to 10% B in 10 min and to 80% in 14 min and was then held for 5 min, before being brought back to the initial conditions and held for 6 min for re-equilibration of the column for the next injection. Capillary voltage was set to 35 V and transfer capillary temperature to 275°C. Measurements were performed in negative mode using the Orbitrap analyzer. Full scan mass spectra (x to y) were generated using 30,000 resolving power and processed and visualized using Xcaliber xx software.

### Imaging on the Ultraflex III® mass spectrometer

A carbon conductive adhesive tape (Plano, Wetzlar, Germany, http://www.plano-em.de) was fixed on an Indium Tin Oxide slide (Bruker Daltonics, Bremen, Germany, http://www.bdal.com) to fix the petals on the slide. The rims of the leave were used for the laser positioning during LDI-MSI. An Ultraflex III® mass spectrometer (Bruker Daltonics) equipped with a Nd∶YAG laser was used for the analysis. All spectra were measured in negative reflectron mode. A raster of 75×75 µm and the large laser focus settings with constant laser intensity and a shot rate of 50 Hz were used. For LDI-MSI of the front side, 9494 raster positions were measured; and 19085 positions for the back side were recorded within a mass range of *m/z* 100–1100. For each raster point, a spectrum was accumulated with 230 laser shots for the front side; and 100 laser shots for the back side; respectively. For image reconstruction, FlexImaging version software version 2.0 (Bruker Daltonics) was used.

### HPLC

HPLC analysis was performed on a Perkin Elmer Series 200 system (Perkin Elmer, Vienna, Austria, http://www.perkinelmer.de) equipped with a photodiode array detector coupled with a 500TR Flow Scintillation Analyzer for the detection of radiolabeled substances according to Chandra *et al.* [45] for assays with okanin and marein as substrates and according to Vande Casteele *et al.* [46] for all other compounds.

### Enzyme preparation

0.5 g plant material, 0.25 g polyclar AT (Serva, Mannheim, Germany, http:www.serva.de) and 0.25 g quartz sand (VWR Vienna, Austria, https://at.vwr.com) were homogenized with 3 mL buffer in a mortar. The homogenate was centrifuged for 10 min at 10,000× g and 4°C. To remove low molecular compounds, crude enzyme preparations were passed through a gel chromatography column (Sephadex G25, GE Healthcare, Freiburg, Germany, http://www.gelifesciences.com). Extraction buffers generally contained 0.4% (v/v) sodium ascorbate (Sigma). For AUS assays, enzyme preparations were liberated from ascorbate during the gel extraction step. Protein content was determined by a modified Lowry procedure [47] using crystalline bovine serum albumine as a standard.

### Enzyme assays

Assays for CHS/CHI, CH3H, DFR, FHT, FNS II and F3′H were performed as described earlier [31], [48].

### Enzyme characterization

All data represents an average of at least three independent experiments. Determination of the pH optimum was carried out as described for the standard AUS assay, but using 0.2 M McIlvaine buffers with pH values between 4.5 and 9.0.

### Standard assay for aurone synthase

The reaction mixture in a final volume of 100 µL contained: 1 nmol [^14^C]butein (150 Bq) or 10 nmol unlabelled chalcones, 3 µg enzyme preparation, 90 µL 0.1 M K_2_HPO_4_/KH_2_PO_4_ buffer pH 7.5. The reaction was started by the addition of the enzyme preparation and the assay was incubated for 15 min at 30°C. The reaction was stopped by addition of 10 µL glacial acetic acid and 70 µL methanol (both VWR). After centrifugation for 10 min at 22,000× g 100 µL of the reaction mixture were subjected to HPLC analysis.

## Supporting Information

Figure S1 **Absorbance spectra of the chalcone butein (full lines) and the aurone sulfuretin (dashed lines) in acidic (red) and alkaline (blue) environment.**
(TIF)

Table S1 **AUS activity in different tissues of** ***B. ferulifolia*.**
(DOC)

Table S2 **Flavonoid and 4-deoxyaurone biosynthesis in the base and apex of the petals of buds and flowers.**
(DOC)
