# Supplementary figures and images for: 4-Deoxyaurone Formation in Bidens ferulifolia (Jacq.) DC
Source: PLoS One. 2013 May 8;8(5):e61766. doi: 10.1371/journal.pone.0061766 (PMC3648546; doi:10.1371/journal.pone.0061766)

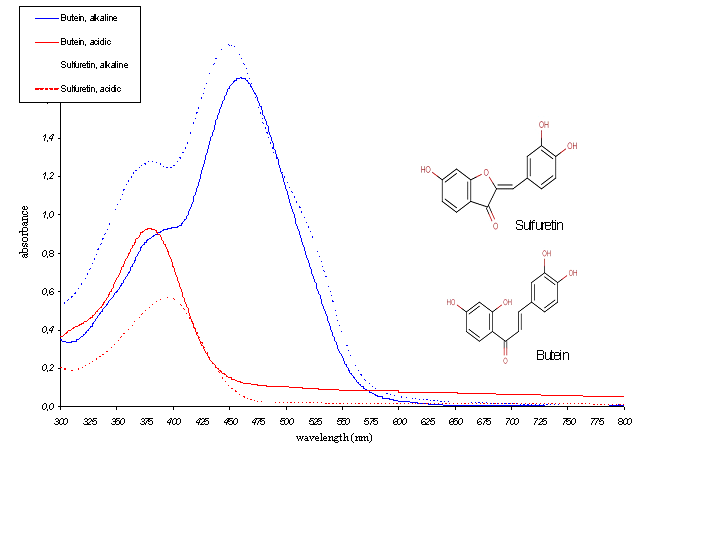

Supplement: Figure S1 — Absorbance spectra of the chalcone butein (full lines) and the aurone sulfuretin (dashed lines) in acidic (red) and alkaline (blue) environment. (TIF) [file pone.0061766.s001.tif]
