# Supplementary material for: 4-Deoxyaurone Formation in Bidens ferulifolia (Jacq.) DC
Source: PLoS One. 2013 May 8;8(5):e61766. doi: 10.1371/journal.pone.0061766 (PMC3648546; doi:10.1371/journal.pone.0061766)
Supplement: Table S1 — AUS activity in different tissues of B. ferulifolia. (DOC) [file pone.0061766.s002.doc]

Table S1: AUS activity in different tissues of *B. ferulifolia*

| **tissue** | **protein concentration (µg/µl)** | **specific activity (µkat/kg protein)** | **activity**  **(µkat/kg FW)** |
| --- | --- | --- | --- |
| petals (ray florets) | 16.7^1^/6.0^2^/6.6^3^ | 2.8^1^/5.7^2^/7.7^3^ | 0.3^1^/0.2^2^/0.3^3^ |
| petals (disc florets) | 10.5 | 1 | 0.06 |
| sepals | 16.3^1^/16.4^2^/15.0^3^ | 3.2^1^/4.6^2^/3.6^3^ | 0.3^1^/0.4^2^/0.3^3^ |
| leaves | 35.0^4^/24.1^5^ | 0.2^4^/0.4^5^ | 0.4^1^/0.5^2^ |
| stems | 7.8 | 1.7 | 0.08 |
| roots | 1.6 | 23.6 | 0.2 |

^1^buds, ^2^opening flowers, ^3^open flowers, ^4^young, ^5^fully developed
