# Supplementary material for: 4-Deoxyaurone Formation in Bidens ferulifolia (Jacq.) DC
Source: PLoS One. 2013 May 8;8(5):e61766. doi: 10.1371/journal.pone.0061766 (PMC3648546; doi:10.1371/journal.pone.0061766)
Supplement: Table S2 — Flavonoid and 4-deoxyaurone biosynthesis in the base and apex of the petals of buds and flowers. (DOC) [file pone.0061766.s003.doc]

Table S2: Biosynthesis of flavonoid and 4-deoxyaurones in the base and apex of the petals of buds and flowers

|  | activities in pkat/g fresh weight | | | | | | |
| --- | --- | --- | --- | --- | --- | --- | --- |
|  | CHS | AUS | FHT | DFR | FNS II | F3’H | CH3H |
| buds base | 25.3 | 41.8 | 0.3 | 0 | 2.0 | 1.8 | 2.5 |
| buds apex | 19.8 | 41.8 | 0.2 | 0 | 0.5 | 2.5 | 2.1 |
| flower base | 12.0 | 46.2 | 0.3 | 0 | 0 | 0.8 | 1.2 |
| flower apex | 4.1 | 49.5 | 0.3 | 0 | 0 | 0.5 | 1.0 |
